# Enhanced biosynthesis of phenazine-1-carboxamide by engineered *Pseudomonas chlororaphis* HT66

**DOI:** 10.1186/s12934-018-0962-3

**Published:** 2018-07-25

**Authors:** Huasong Peng, Pingyuan Zhang, Muhammad Bilal, Wei Wang, Hongbo Hu, Xuehong Zhang

**Affiliations:** 1 State Key Laboratory of Microbial Metabolism, School of Life Sciences and Biotechnology, Shanghai Jiao Tong University, 800 Dongchuan Road, Shanghai, 200240 People’s Republic of China; 2 School of Life Science and Food Engineering, Huaiyin Institute of Technology, Huaian, 223003 China; 3 National Experimental Teaching Center for Life Sciences and Biotechnology, Shanghai Jiao Tong University, Shanghai, 200240 China

**Keywords:** *Pseudomonas chlororaphis*, Phenazine-1-carboxamide, Gene inactivation, Phenazine regulation

## Abstract

**Background:**

Phenazine-1-carboxamide (PCN), a phenazine derivative, is strongly antagonistic to fungal phytopathogens. The high PCN biocontrol activity fascinated researcher’s attention in isolating and identifying novel bacterial strains combined with engineering strategies to target PCN as a lead molecule. The chemical route for phenazines biosynthesis employs toxic chemicals and display low productivities, require harsh reaction conditions, and generate toxic by-products. Phenazine biosynthesis using some natural phenazine-producers represent remarkable advantages of non-toxicity and possibly high yield in environmentally-friendlier settings.

**Results:**

A biocontrol bacterium with antagonistic activity towards fungal plant pathogens, designated as strain HT66, was isolated from the rice rhizosphere. The strain HT66 was identified as *Pseudomonas chlororaphis* based on the colony morphology, gas chromatography of cellular fatty acids and 16S rDNA sequence analysis. The secondary metabolite produced by HT66 strain was purified and identified as PCN through mass spectrometry, and ^1^H, ^13^C nuclear magnetic resonance spectrum. The yield of PCN by wild-type strain HT66 was 424.87 mg/L at 24 h. The inactivation of *psrA* and *rpeA* increased PCN production by 1.66- and 3.06-fold, respectively, which suggests that *psrA* and *rpeA* are PCN biosynthesis repressors. *q*RT-PCR analysis showed that the expression of *phzI*, *phzR*, and *phzE* was markedly increased in the *psrA* and *rpeA* double mutant than in *psrA* or *rpeA* mutant. However, the transcription level of *rpeA* and *rpeB* in strain HT66Δ*psrA* increased by 3.52- and 11.58-folds, respectively. The reduced *psrA* expression in HT66Δ*rpeA* strain evidenced a complex regulation mechanism for PCN production in HT66.

**Conclusion:**

In conclusion, the results evidence that *P. chlororaphis* HT66 could be modified as a potential cell factory for industrial-scale biosynthesis of PCN and other phenazine derivatives by metabolic engineering strategies.

**Electronic supplementary material:**

The online version of this article (10.1186/s12934-018-0962-3) contains supplementary material, which is available to authorized users.

## Background

During the past couple of decades, plant growth-promoting rhizobacteria (PGPR) have attracted a great deal of researcher’s attention in the field of agriculture. Majority of these bacteria function as biological control agents in various plant-pathogen systems and promote plant growth by protecting them from various soil-borne pathogens [1, 2]. The secondary metabolites produced by PGPR species, such as phenazine derivatives, play a noteworthy role in interactions between these strains and other organisms [3–5]. Phenazines are nitrogen-containing heterocyclic pigments that exhibit broad-spectrum antifungal, antibacterial, anti-tumor, antimalarial, and antiparasitic potentialities in various niches [6–9]. For example, phenazine-1-carboxylic acid (PCA) greatly reduces the risk of a severe wheat root disease caused by *Gaeumannomyces graminis* var. *tritici* [10, 11]. Phenazine-1-carboxamide (PCN) possesses notable antifungal activity against *Fusarium oxysporum* f. sp. *radicis*-*lycopersici* [12, 13]. In addition, phenazine derivatives show great potential for use as electron acceptors and donors [14], components of microbial fuel cells (MFC) [15], and environmental sensors and biosensors [16, 17].

Most phenazine producing *Pseudomonas* strains are isolated from soil environment [6, 18]. *Pseudomonas* can synthesize several phenazine compounds, such as PCA produced by *P. aeruginosa* [19], and 2-hydroxy-phenazine (2-OH-PHZ) produced by *P. chlororaphis* [20]. The hypothesis that PCA is transformed further into PCN by the glutamine amidotransferase enzyme PhzH, was first described in *P. chlororaphis* PCL1391 [21]. The high biocontrol activity of PCN against many fungal plant pathogens fascinated researcher’s interest in isolating and identifying novel bacterial strains together with engineering strategies to target PCN as a lead molecule [3, 22].

*Pseudomonas* species utilize combinations of conserved regulatory systems for phenazine biosynthesis in response to diverse environmental signals. These regulatory elements include the *gacS*/*gacA* and *rpeA/rpeB* two-component signal transduction, the *tetR* homolog *psrA, pip* (*p*henazine *i*nducing *p*rotein), alternative sigma factor *rpoS*, and the quorum sensing system *phzI*/*phzR* [23]. *GacS* and *gacA* function as master regulators in *Pseudomonas* species [24]. The membrane-bound histidine kinase, *GacS* activates the cytoplasmic response regulator by phosphorylation at the N-terminal domain of *GacA* responding to environmental signals. As a global factor, *rpoS* controls a series of quorum-sensitive gene expression at the onset of stationary phase, depending on the growth conditions and species [25]. *psrA*, which responses to cell density and controls *rpoS* gene promoter [26], represses or stimulates *N*-acyl homoserine lactones (AHL) synthesis as well as PCN production depending on the growth medium in *P. chlororaphis* PCL1391 [27, 28]. Situated at downstream of *psrA*/*rpoS* and upstream of *phzI*/*phzR*, *pip* is essential for phenazine production and shows similarity to members of the *tetR/acrR* family of transcriptional regulators [29]. Autoinducers produced by *phzI* are supposed to bind to *PhzR*. The complex subsequently switches on expression of the *phz* operon which is involved in phenazines biosynthesis. Thus, the phenazine regulatory genes ultimately act on the quorum-sensing system [30]. *rpeA*/*rpeB* is another regulatory element that independently regulates phenazine biosynthesis using *pip* as a common regulatory intermediate with *rpoS* [31]. Generally, synthesis of the core phenazine PCA is encoded by a conserved cluster of several genes in phenazine-producing bacteria [32]. However, few studies have focused on the effects of *rpeA* in regulating PCN production and the connection between *rpeA* and *psrA* in phenazine regulatory network [28, 31].

The present study reports the isolation of a novel *Pseudomonas* strain, designated as HT66; from the rhizosphere soil of rice. An antimicrobial compound was separated from organic fraction and purified by preparative high-performance liquid chromatography (HPLC). The compound was identified as PCN with the highest production of 424.87 mg/L by the wild-type strain. The effects of the deletion of *psrA* and *rpeA* on phenazine biosynthesis in *P. chlororaphis* HT66 were investigated by constructing single mutants (HT66Δ*psrA* and HT66Δ*rpeA*) as well as the double mutant (HT66Δ*psrA*Δ*rpeA*). We speculated a regulating cascade that *psrA* regulates the phenazines biosynthetic operon by quorum sensing independent regulators *rpeA*/*rpeB* in *P. chlororaphis* HT66.

## Methods

### Bacterial strains, media, and cultivation conditions

CFC medium (containing cephaloridine, Fucidin, and cetrimide) purchased from the Qingdao Haibo was used to isolate pseudomonads from the soil samples [33]. The pseudomonad isolate and its mutants were cultivated on King’s B medium (KB) at 28 °C using standard methods and preserved in 30% (v/v) glycerol at − 80 °C for long-term maintenance. *Escherichia coli* DH5α was grown on Luria–Bertani (LB) medium (tryptone 10.0 g, yeast extract 5.0 g, NaCl 10.0, 15 g/L agar if solid medium). If needed, the following antibiotics were added to the medium at following concentrations: tetracycline (Tc, 150 μg/mL) and gentamicin (Gm, 40 μg/mL) for *Pseudomonas*; spectinomycin (Sp, 100 μg/mL) and Tc (20 μg/mL) for *E. coli*. The plasmid pK18mobsacB used to harbor homologous fragments with the target a sequence deriving from pK18 is a broad-host-range vector containing genetic loci *sacB* and Km^r^ (Accession No. FJ437239) [34]. LB broths supplemented with 15% sucrose were used to counter select the suicide plasmid pEX18Tc. The bacterial strains, plasmids, and primers used in this study are listed in Table 1.

**Table 1:** Bacterial strains, selected plasmids, and primers used in this study

Strain, plasmid or primer target gene	Relevant characteristics	Reference or source
*Escherichia coli* strains		
DH5α	*supE44 ∆lacU169* (*ϕ80 lacZ∆M15*) *recA hsdR17 recA1 endA1 gyrA96 thi*-*1 relA*-*1*	Invitrogen
SM10	*thi*-*1 thr leu tonA lacY supE recA:: RP4*-*2 tc:: Mu; lambda pir, Kan*^*R*^	Invitrogen
*Pseudomonas chlororaphis* strains		
HT66	Wild-type, *Sp*^*R*^	This study
Δ*psrA*	Mutant of HT66 in the *psrA* gene, *Kan*^*R*^	This study
Δ*rpeA*	Mutant of HT66 in the *rpeA* gene, *Gm*^*R*^	This study
Plasmids		
pEX18Tc	Sucrose sensitive cloning vector, *Tc*^*R*^	TAKARA
pEX-*psrA*	*psrA* from HT66 in pEX18Tc, *Tc*^*R*^	This study
pEX-*psrA*-*Kan*^*R*^	*Kan*^*R*^ cassette insertion from pBS(kan) in pEX-*psrA*; *Tc*^*R*^,*Kan*^*R*^	This study
pEX-*rpeA*-*aacC1*	*Gm*^*R*^ cassette insertion from pUCGM in pEX-*rpeA*; *Tc*^*R*^,*Gm*^*R*^	Huang et al. [38]
Primer target gene	Primer sequences (5′–3′)	
*psrA*	*psrA*1: TTAAGCTTACGGTCGGGTCGTCGCTGCATA	*Hin*dIII
	*psrA*2: AAGAGCTCCGCTGTTCATGCCACGGATAAAG	*Sac*I
*rpeA*	*rpeA*E1: TATTAATCTAGACCTGTTCAGCCGTTCCGAAT	*Xba*I
	*rpeA*E2: AATTATGAATTC-CCACGCCCAGTTGATCCT	*Eco*RI
*Kan*^*R*^ (kanamycin resistance gene)	pBS-Kan-R: TATAGCATGCTCAACCGTGGCTCCCTCA	*Sph*I
	pBS-Kan-F: TTAGCATGCCTTACTGTCATGCCATCCG	*Sph*I
*aacC1* (gentamycin resistance gene)	Gm-R and Gm-F: AGAATCGATATCCCCGGGTACCGAG	*Cla*I

^a^*Kan*^*R*^, *Sp*^*R*^*, Amp*^*R*^, *Gm*^*R,*^ and *Tc*^*R*^ represent kanamycin, spectinomycin, ampicillin, gentamycin and tetracycline resistance, respectively

### Isolation of phenazine-producing pseudomonads

The rhizosphere soil of rice was used for pseudomonad isolation. The sampling site was located in Jinhui Town (Shanghai City, China) and the soil core was clayey, pH 7.0–7.8. Rice roots, together with soil, were collected and pretreated following a previously described method [35]. The cores were suspended in phosphate buffer solution (120 mM NaCl and 2.7 mM KCl in 10 mM phosphate buffer, pH 7.6) by vortex mixing and sonication in an ultrasonic cleaner, followed by centrifugation at 3000 rpm. A 5 mL of the resulting supernatant was then inoculated in 100 mL CFC-selective medium in a 250-mL conical flask. The mixture was cultured overnight at 28 °C at an agitation speed of 180 rpm, and 200 μL mixtures were spread onto each CFC-selective agar plate at the proper dilutions (20–200 colonies per Petri dish) after the designated incubation period.

### 16S rDNA sequence and phylogenetic analysis

The 16S rDNA of strain HT66 was amplified from genomic DNA by a polymerase chain reaction (PCR) using the forward primer 27F (5′-AGAGTTTGATCMTGGCTCAG-3′) and reverse primer 1492R (5′-TACGGHTACCTTGTTACGACTT-3′) [36]. PCR was performed using the initial denaturation at 94 °C for 3 min, 30–35 cycles at 94 °C for 30 s, at annealing temperature 50 °C for 1 min, at 72 °C for 2 min extension, and a final polymerization at 72 °C for 5  min. The PCR-amplified products were sequenced by Beijing Genomics Institute (BGI, China). A phylogenetic tree was constructed based on the dissimilarity index by comparing with validly published 16S rDNA sequences of related type strains from GenBank, using the neighbor-joining method and ClustalX (version 1.83) program to perform multiple alignments. The phylogenetic and molecular evolutionary analyses were carried out using MEGA version 5.0 [37].

### Analysis of whole cell fatty acid

After 24 h incubation on KB, 40 mg of bacterial cells from the KB medium were harvested. Saponification, methylation, extraction, and base wash were conducted according to previously described methods to convert the total cellular fatty acids to fatty acid methyl esters (FAME) for gas chromatography (GC) analysis. An Agilent 7890A GC system equipped with a capillary-optimized flame ionization detector (FID) and an ARC Polyarc reactor (PA-RRC-A02) was used for the analysis of FAME using helium (99.999%, Praxair) as the carrier and FID makeup gas. The GC results were automatically compared with Sherlock libraries by a covariance matrix, principal component analysis, and pattern recognition software to identify bacteria based on their distinct fatty acid profiles.

### Purification and identification of active compounds

An initial culture of strain HT66 was grown in KB liquid medium for 24 h at 28 °C (180 rpm). The culture was transferred to 20-L KB medium in a bioreactor (Sartorius & BIOSTAT C+) at 1.0% inoculation ratio. Cultivated for 3 days, the fermented broth was extracted with equal volume of ethyl acetate three times. Finally, a 10 g crude extract was harvested after the organic phase was evaporated under vacuum pressure at 37 °C. The concentrated crude metabolites were dissolved in methanol, and major compounds were purified by preparative HPLC (Shimadzu LC8A). The molecular structures of the active fractions were elucidated by mass spectrum, ^1^H, and ^13^C nuclear magnetic resonance (NMR) spectrometer.

Ultra-performance liquid and quadrupole time-of-flight mass spectrometry (UMS) was performed on ACQUITY™ UPLC & Q-TOF MS Premier (Waters, USA). The instrument was equipped with electrospray ionization to separate peaks that cannot be resolved by LC alone and time of flight mass spectrometers in mass detection. ^1^H and ^13^C NMR spectra were acquired on a Bruker model Avance III 400 MHz (BRUKER, Switzerland) spectrometer at 400 MHz for ^1^H and 100 MHz for ^13^C in MeOD as a solvent.

### Construction of *psrA*, *rpeA*, and *psrA*/*rpeA* double mutants in strain HT66

Gene deletions in *P. chlororaphis* HT66 strain were conducted using a markerless deletion method with pK18mobsacB as described earlier [38]. The primers *psrA*1 and *psrA*2 were designed to amplify the gene *psrA* from strain HT66 at the annealing temperature 47.4 °C, whereas primers pBS-Kan-R and pBS-Kan-F were used to amplify the Kan resistance gene *Kan*^*R*^ in plasmid pBS(Kan) at 50 °C. The purified PCR-amplified products of *psrA* were cloned into the suicide plasmid pEX18Tc and inserted by the fragment containing *Kan*^*R*^ at a filled *Sph*I site. The resulting plasmid pEX18Tc containing 2.5 kb *Hin*dIII–*Sac*I fragment was then mobilized from *E. coli* SM10 to strain HT66 by biparental mating. Putative transconjugants were screened on LB plates supplemented with 15% sucrose, Sp, and Kan. After the second crossing over, Kan-resistant, Tc-sensitive, and sucrose-resistant recombinants were recovered. Sequencing analysis revealed that the *psrA* gene open reading frame was inserted by Kan resistant gene approximately 491 bp downstream of the start codon. The resultant chromosomally inactivated *psrA* mutant was designated as HT66Δ*psrA*.

The construction of *rpeA* inactive and *psrA*/*rpeA* double inactive mutants was carried out using the same methodology described previously for the *psrA* mutant.

### Analysis of PCA and PCN

A total of 0.5 mL of fermented broth was firstly adjusted to pH 2.0 using 6 M HCl and extracted with 3 mL of ethyl acetate by vigorous shaking. After centrifugation, the organic phase (0.2 mL) containing phenazine compounds were evaporated under vacuum pressure. The resulting residue was dissolved in methanol, filtered through an organic phase filter (0.22 μm), and subjected to HPLC (Model 1260 infinity, Agilent Technologies, Santa Clara, USA) using a WondaSil C18-WR reversed-phase column (5 μm; 4.6 × 250 mm, Shimadzu, Japan) at 254 nm. The mobile phase changed during the detection process i.e. methanol: ammonium acetate = 20:80 (v/v) in the first 5 min, then changing to 50:50 in 5–25 min and 20:80 again in the last 5 min (25–30 min). The flow rate was 1.0 mL/min, and the column temperature was maintained to 30 °C. The retention times for PCA and PCN were approximately 9.523 and 17.217 min, respectively (Additional file 1: Fig. S1).

### RNA extraction and qRT-PCR

After incubation for 72 h, HT66 cells were immediately harvested by centrifugation (at 12,000  rpm for 2 min at 4 °C), and the total RNA was extracted using RNAprep Pure Cell/Bacteria Kit (TIANGEN, China). Genomic DNA was eliminated using DNase. cDNA was synthesized using random primers at 37 °C for 1 h with Quantscript RT Kit (TIANGEN, China). The qRT-PCR was performed in 20 μL volume with aliquots (1 μL) of cDNA or water (no-template control), primers (200 nM final concentration), and 10 μL of SuperReal PreMix Plus (SYBR green I, TIANGEN, China) in an Eppendorf RT-PCR system. Primers were designed to amplify 148–152  bp fragments (Additional file 1: Table S1). The qPCR amplifications were carried out at 95 °C for 10 min, followed by 40 cycles of 95 °C for 15 s, 45 °C for 30 s, and 68 °C for 15 s, and a final dissociation curve analysis step from 68 to 95 °C.

Amplification specificity for each reaction was verified by melting-curve analysis according to the Eppendorf RT-PCR system software. Threshold cycle (Ct) values were used to calculate relative fold changes in expression of each gene by the ΔΔCt analysis [39]. The *rpoD* gene served as a reference gene to normalize each gene expression [32]. The primer pairs used to detect the gene expression of *gacA*, *gacS*, *psrA*, *rpoS*, *pip*, *rpeA*, *rpeB*, *phzR*, *phzI,* and *phzE* in strain HT66 wild-type, HT66Δ*psrA*, HT66Δ*rpeA*, and HT66Δ*rpeA*Δ*psrA* are portrayed in Additional file 1: Table S1.

### Nucleotide sequence accession numbers

Nucleotide sequences of the 16S rDNA/*phzH*/*rpeA* and *psrA* gene of *P. chlororaphis* HT66 were deposited in GenBank. The Accession Numbers are KF857481/KF900067/KF900068 and KF900069.

## Results

### Identification of *P. chlororaphis* HT66

**Fig. 1:**
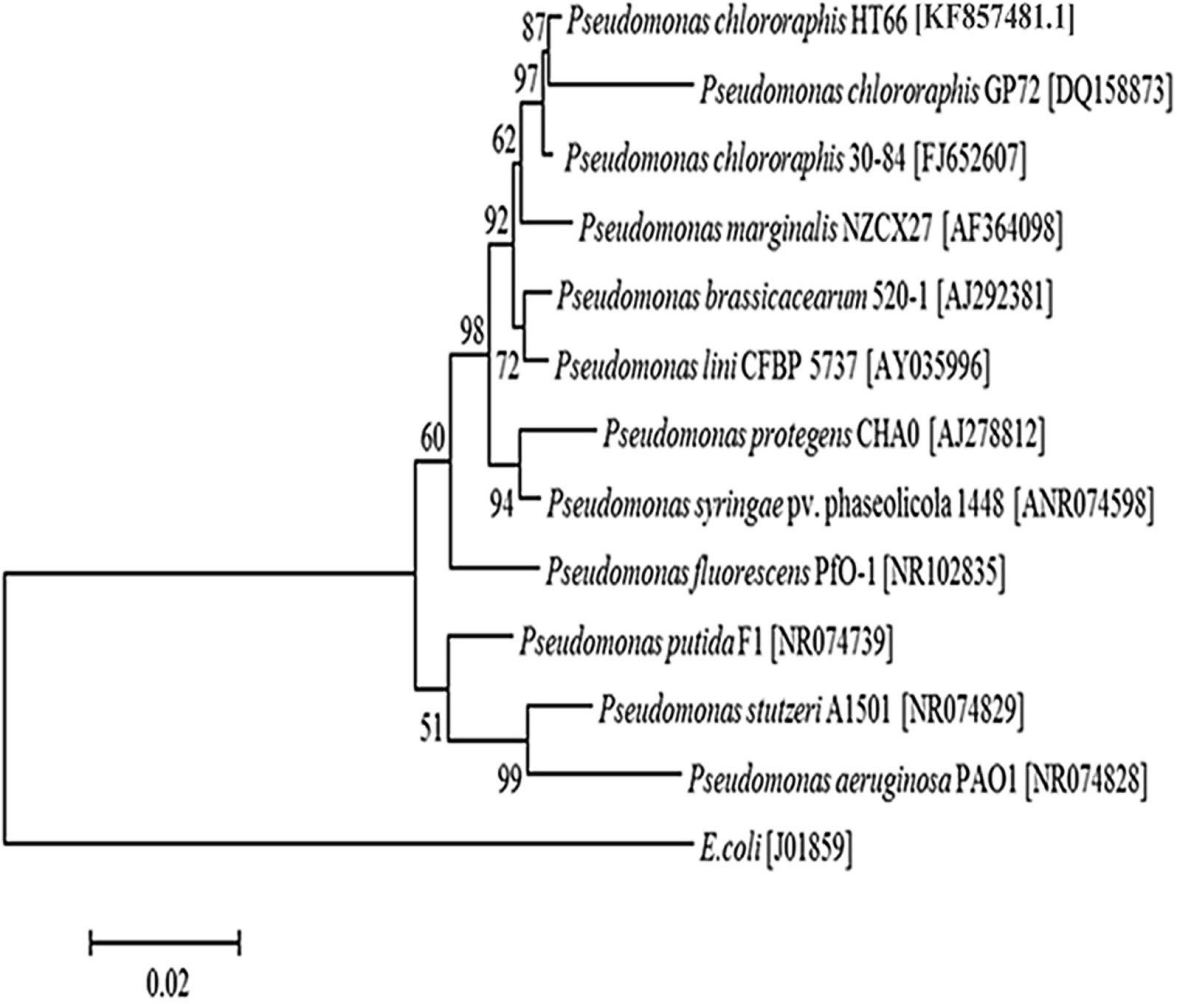
Phylogenetic tree of *Pseudomonas chlororaphis* HT66 and its related *Pseudomonas* species based on 16S rDNA sequences. The phylogenetic tree was constructed based on the percent difference in genetic relationships between the allied strains in the National Center for Biotechnology Information (NCBI) database. The length of each pair of branches represents the distance between nucleotide sequence pairs, whereas units at the top of the tree indicate the percentage of similarity among these aligned sequences (28, 29, 31)

The strain HT66 exhibited notable antifungal abilities against *Rhizoctonia solani* and *Pythium ultimum*, the pathogen of Stevia leaf spot disease, and *Fusarium oxysporum* f. sp. *niveum* (Additional file 1: Fig. S2). The colony of strain HT66 on KB plate was circular and moist with light yellow coloration after incubation of 24 h and with a dark green pigment on the colony surface at 72 h or longer. The phylogenetic tree is one of the major criteria of microbial taxonomy. A 1407 bp nucleotide sequence of the 16S rDNA gene was amplified from strain HT66 by universal primers 27F/1492R. The phylogenetic tree based on 16S rDNA indicates that strain HT66 is close to *P. chlororaphis* with 99% similarity (Fig. 1). The report of fatty acid methyl ester analysis by Sherlock MIS database showed that the strain HT66 was at the highest Sim index to *P. chlororaphis* (Additional file 1: Fig. S3, Table S2). Based on above experiments, we concluded that strain HT66 belongs to *P. chlororaphis* species (Strain HT66 was preserved in China Center for Type Culture Collection at Wuhan, China. Accession No. is CCTCC M 2013467).

### Structural identification of the antifungal compound

**Fig. 2:**
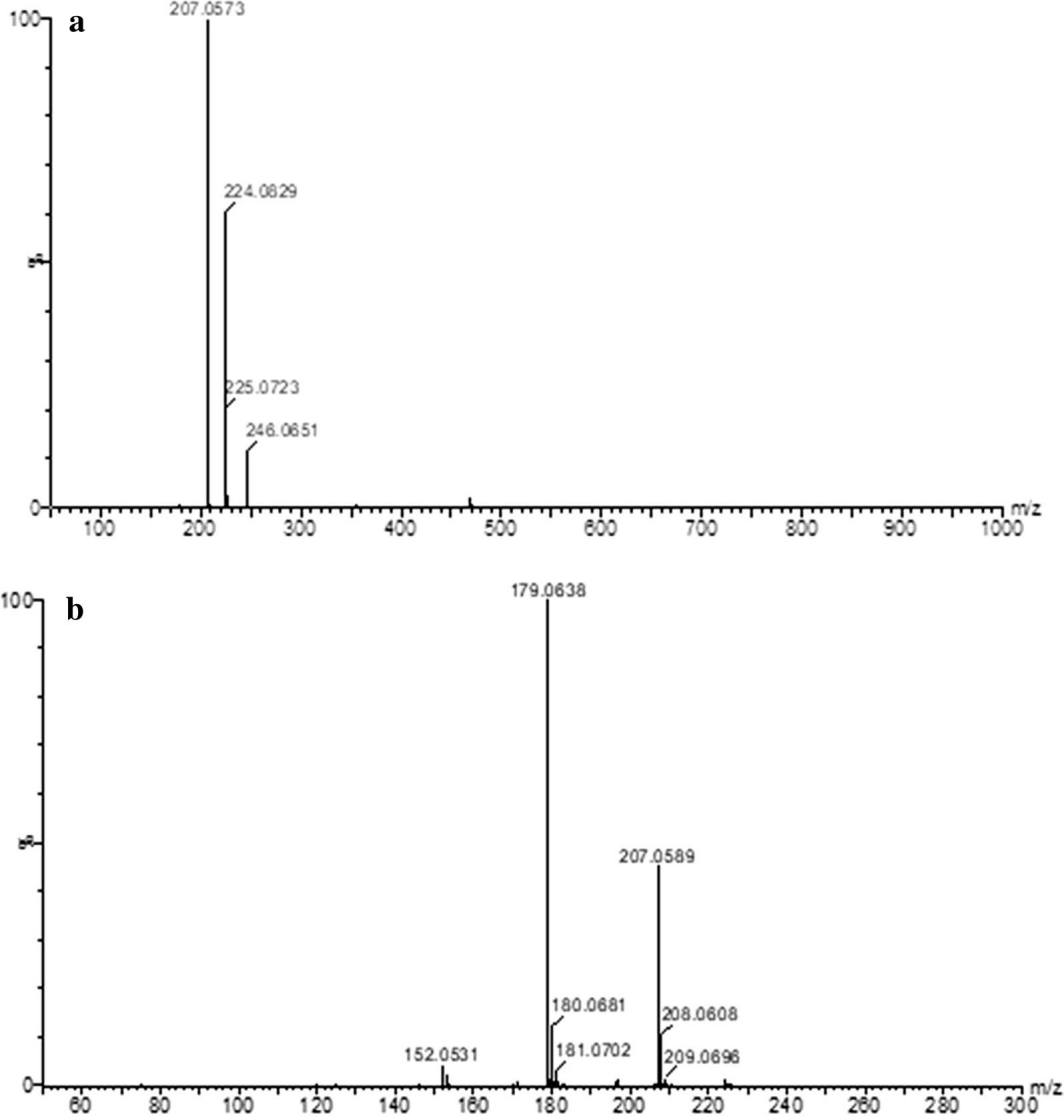
Mass spectrum of the antifungal metabolite produced by *Pseudomonas chlororaphis* HT66, **a** time-of-flight mass spectrometry of purified compound of strain HT66, and **b** MS/MS spectrometry of the molecular weight 224.08 compound

**Fig. 3:**
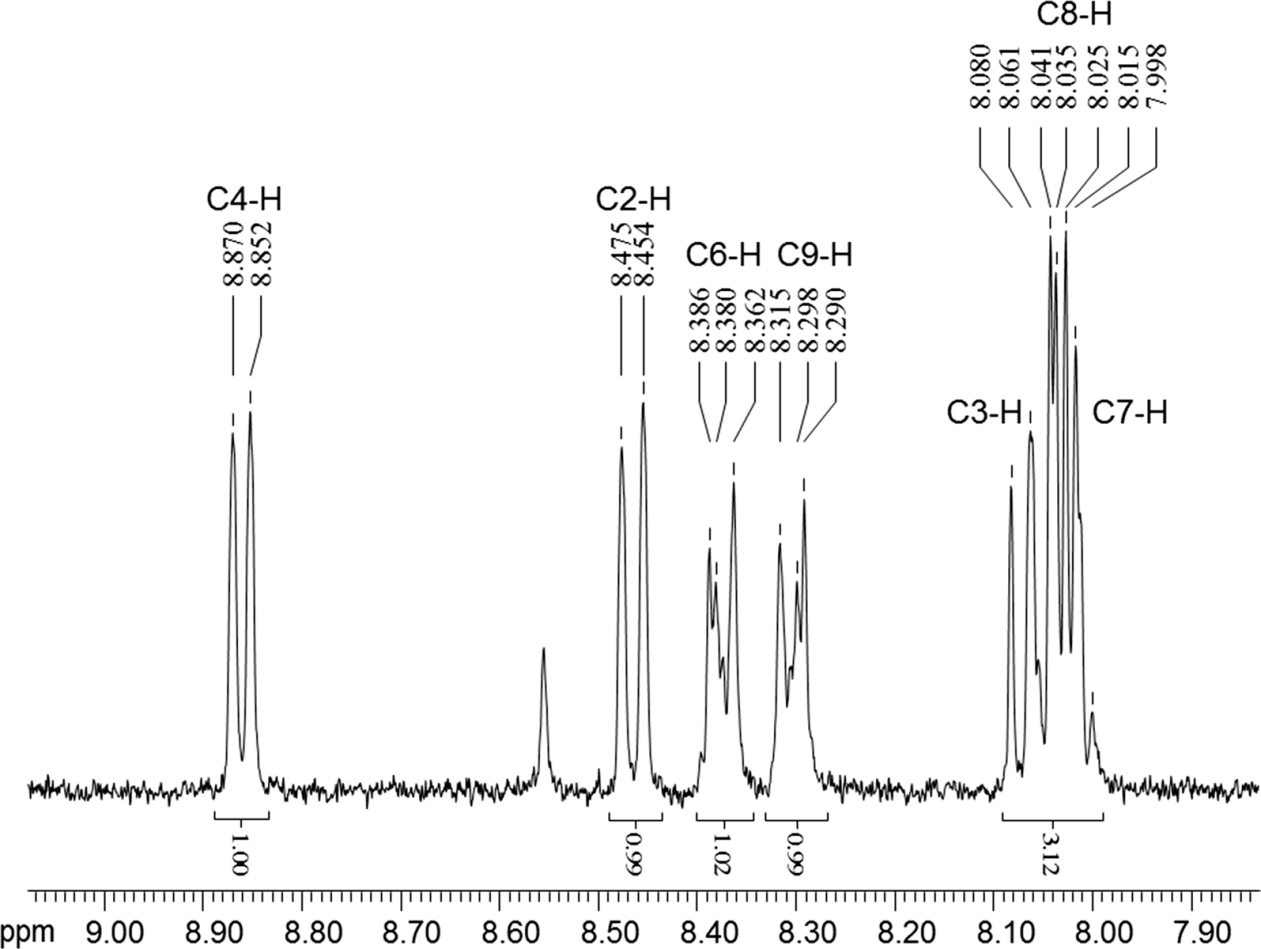
^1^H NMR signals from the antifungal metabolite produced by *Pseudomonas chlororaphis* HT66

**Fig. 4:**
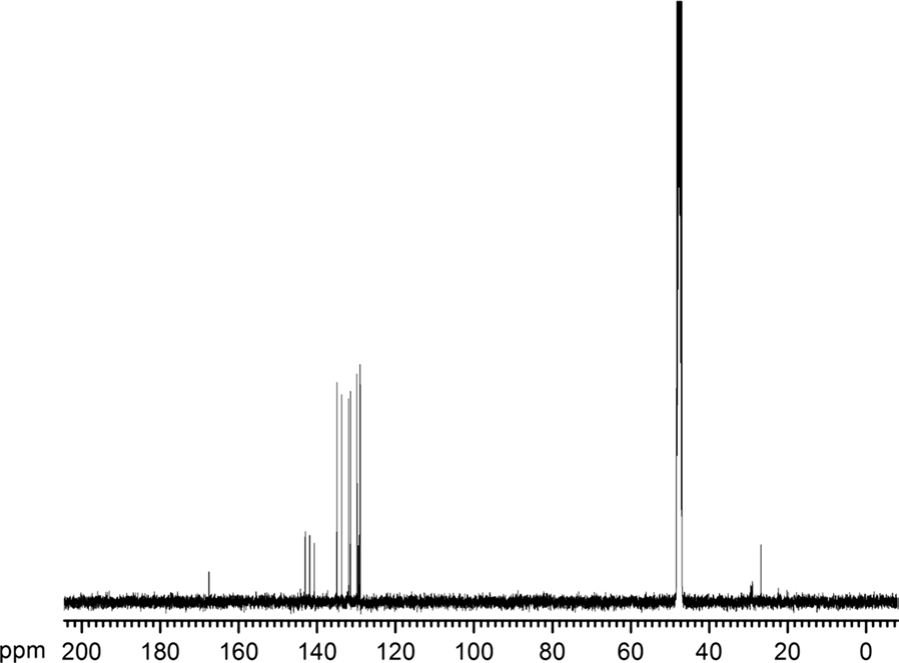
^13^C NMR signals from the antifungal metabolite produced by *Pseudomonas chlororaphis* HT66

From mass spectrum at the low cone voltage, the molecular mass of the antifungal compound on UMS was 224.08 as *m/z* 225.07 corresponding to the protonated [M + H]^+^, *m/z* 246.06 corresponding to the sodiated [M + Na]^+^, and *m/z* 207.06 corresponding to the loss of NH_3_, as shown in Fig. 2. In the high cone voltage spectrum, a very intense fragment ion was observed at *m/z* 179.06, corresponding to the eliminated carboxamide functional group and protonated phenazine ring. ^1^H NMR spectrum displayed signals corresponding to protons in the vicinity of heteroatoms, which are very useful to disclose chemical structure (Fig. 3). The ^1^H NMR spectrum chemical shifts of the purified compound of *P. chlororaphis* HT66 were as follows: ^1^H NMR (MeOD, 400 MHz, ppm), H-2 (d 8.46, dd), H-3 (d 8.07, dd), H-4 (d 8.86, dd), H-6 (d 8.38, ddd), H-7 (d 8.00, ddd), H-8 (d 8.02, ddd), and H-9 (d 8.29, ddd). The ^13^C NMR spectrum of the purified antifungal compound exhibited the appearance of a carbonyl carbon of the amide group at d 167.53 (Fig. 4). The residual aromatic carbons showed signals between d 128.93 and 143.06. Based on the combination of UMS and NMR data, the purified compound isolated from *P. chlororaphis* HT66 was definitively identified as a PCN.

### Effect of *psrA* and *rpeA* on phenazine production

**Fig. 5:**
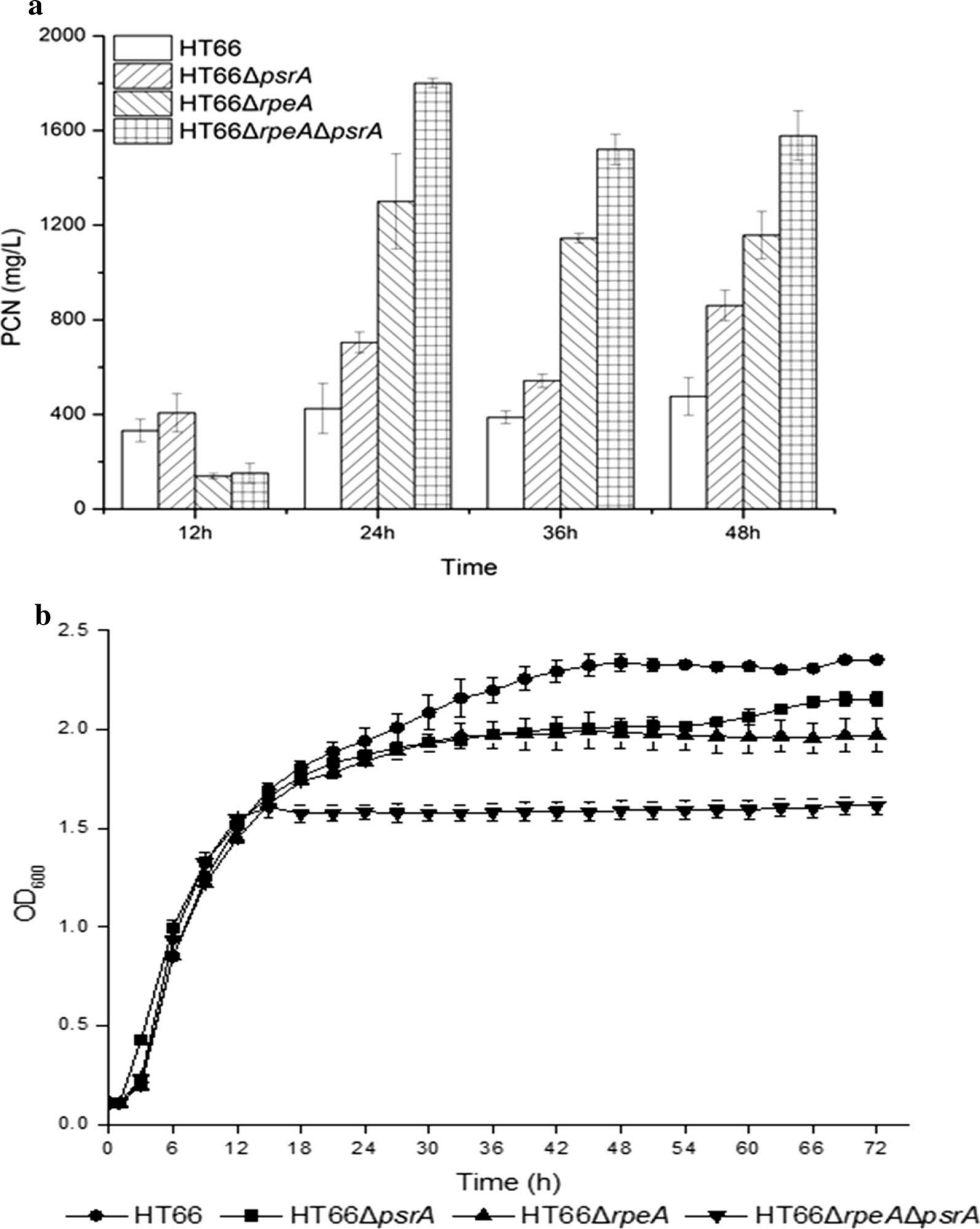
**a** Cell growth, and **b** phenazine-1-carboxamide production of *Pseudomonas chlororaphis* HT66 and its derivative strains (HT66—*P. chlororaphis* HT66 wild-type strain; HT66*ΔpsrA*—*psrA* in-frame deletion mutant of HT66; *HT66ΔrpeA*—*rpeA* in-frame deletion mutant of HT66; *HT66ΔpsrAΔrpeA*—*psrA* and *rpeA* in-frame deletion mutant of HT66)

In this study, strain HT66Δ*psrA,* HT66Δ*rpeA*, and HT66Δ*psrA*Δ*rpeA* were constructed and subjected to fermentation. Time-course fermentation pro-file of PCN production and cell growth are portrayed in Fig. 5. Experimental results showed that the production of PCN substantially increased in all the mutant strains (Fig. 5a). After fermentation of 24 h, the production of PCN in the wild-type HT66 was 424.87 mg/L and increased to 703.93 mg/L in strain HT66Δ*psrA*, a 1.66-fold increase, representing the repressive effect of *psrA* on phenazines biosynthesis in strain HT66. Similarly, compared with wild-type HT66, the production of PCN increased 3.06-fold in strain HT66Δ*rpeA*, which was as high as 1300.69 mg/L. Moreover, the PCN yield increased to 1800.54 mg/L (4.24-fold increase) in double mutant strain HT66Δ*psrA*Δ*rpeA* in comparison with the wild-type HT66. The results demonstrated that both *psrA* and *rpeA* play an important role in the regulation and biosynthesis of phenazines in strain HT66. The cell density of HT66Δ*psrA* and HT66Δ*rpeA* strains was modestly decreased, whereas a marked decline in cell density was observed in case of HT66Δ*psrA*Δ*rpeA* in comparison with the wild-type HT66 strain (Fig. 5b).

### Relative gene transcription levels

As described earlier, a series of regulatory genes are involved in phenazines metabolism cascade. qRT-PCR experiments were carried out to measure the expression of related genes to elucidate the action of *psrA* and *rpeA* for phenazines regulatory network in *P. chlororaphis* HT66 strains. The target genes are homologous of known PCA/PCN regulating genes and have direct or indirect interaction with *psrA*/*rpeA* in other bacterial species. Table 2 illustrates the relative fold changes in expression of all the tested genes. Though the *phzR* expression difference in wild-type and the *psrA*/*rpeA* mutant was not significant, *phzI* expression was markedly high, especially in the double mutant. Consistent with phenazine production, *phzE* expression was increased 3.33-fold compared to the wild type in HT66Δ*psrA*, 4.15-fold in HT66Δ*rpeA*, and 16.53-fold in HT66Δ*psrA*Δ*rpeA*. On the contrary, the expression of *phzI*, *phzR,* and *phzE* was amplified much more in HT66Δ*psrA*Δ*rpeA* than in HT66Δ*psrA* and HT66Δ*rpeA* which indicated that knockout of *psrA* and *rpeA* can improve the productivity of PCN.

**Table 2:** Relative fold change of gene expression in *psrA/rpeA* and double mutant

Gene	Fold change in expression^a^			Description^b^
	HT66Δ*rpeA*	HT66Δ*psrA*	HT66Δ*rpeA*Δ*psrA*	
*gacA*	1.24 (0.72 to 1.75)	2.78 (2.09 to 3.68)	1.19 (− 1.17 to 1.67)	Response regulator
*gacS*	2.08 (1.78 to 2.37)	1.17 (0.71 to 1.69)	1.48 (1.04 to 2.1)	Sensor protein
*rpeA*	–	*3.52* (2.75 to 5.04)	–	Sensor kinase
*rpeB*	2.39 (1.83 to 2.94)	*11.58* (8.27 to 16.22)	5.06 (4.96 to 6.56)	Response regulator
*psrA*	− 3.82 (− 3.49 to − 4.15)	–	–	*Pseudomonas* sigma regulator
*rpoS*	1.06 (0.68 to 1.43)	1.53 (1.21 to 1.93)	− 1.13 (− 1.58 to 1.23)	RNA polymerase sigma factor
*pip*	4.43 (3.78 to 5.08)	1.46 (1.14 to 1.88)	3.56 (2.58 to 4.9)	Phenazine-inducing protein
*phzI*	10.01 (9.71 to 10.31)	2.73 (2.55 to 2.93)	29.65 (21.55 to 40.79)	Autoinducer synthase
*phzR*	1.03 (0.43 to 1.63)	− 1.06 (− 1.29 to 1.14)	6.06 (4.24 to 8.67)	Transcriptional activator protein
*phzE*	*4.15* (3.44 to 4.86)	*3.33* (3.09 to 3.59)	*16.53* (11.96 to 22.83)	Phenazine biosynthesis protein

^a^Fold change in expression of *gacA/gacS/psrA/rpoS/pip/rpeA/rpeB/phzI/phzR/phzE* genes in *psrA/rpeA* and double mutant compared with the HT66 strain. The gene *rpoD* was used as the reference. Cells were grown in KB for 15 h. These experiments were repeated at least three times, and similar results were obtained. Statistical analysis of ΔΔCt values confirmed the results for each gene tested. Italicface numbers are statistically, significantly different from gene expression, at least 2.0-fold different

^b^Putative function was assigned by similarity to proteins in public databases

We also scrutinized the expression of several phenazine regulatory genes, including *rpoS*, *gacA*/*gacS,* and *pip*, which are known to possess key roles in the biosynthesis of phenazine. Notably, loss of *psrA* resulted in a significantly (*P* < 0.05) increased expression of *rpeA* and *rpeB*, whereas *rpoS* and *pip* expression levels were slightly enhanced. Similarly, the *rpeA* inactivation considerably increased (*P* < 0.05) the transcription of *rpeB* and *pip* but did not affect the transcription of *rpoS*. The results were consistent with those in *rpeA*/*rpeB* and *rpoS* where the system independently regulates phenazine via *pip* in strain 30–84.^31^ In conclusion, qRT-PCR analyses revealed that the inactivation of *psrA* led to a significantly increased expression of *rpeA*, while inactivation of *rpeA* resulted in decreased *psrA* expression levels.

## Discussion

Considering the culture characteristics, phylogenetic analysis of 16S rDNA, and gas chromatography of cellular fatty acids, strain HT66 was identified as *P. chlororaphis*. The *P. chlororaphis* plays an important role in the biological control of plant disease caused by phytopathogens by producing a unique set of secondary metabolites such as PCA, PCN, 2-hydroxy-PCA, and 2-OH-phenazine [4]. Depending on environmental conditions, the number and types of phenazines produced by an individual bacterial strain may also fluctuate. Phenazine derivatives can be synthesized simultaneously or individually in any organism. For example, *P. chlororaphis* GP72 produces PCA accompanied by a small amount of 2-OH-phenazine, whereas *P. chlororaphis* PCL1391 produces mainly PCN.

Based on the results obtained from UMS and NMR, the active metabolite that mediates the broad-spectrum antifungal phenazine derivatives produced by strain HT66 has been chemically characterized as PCN (Figs. 2, 3, 4). In previous studies, the PCN has been identified in *P. chlororaphis* PCL1391, *P. aeruginosa* PAO1 [40], *P. aeruginosa* PUPa3 [41], *Pseudomonas* sp. PUP6 [42], and *P. aeruginosa* MML2212 [13]. The yields of PCN in these strains are too low to be applied for the agricultural purposes. *P. chlororaphis* HT66 can be used as an effective biological control candidate against fungal pathogens, as the highest production of PCN in wild-type strain was recorded to be 424.87 mg/L. Considering in view that PCN displays more potent antifungal activities than PCA in wider pH ranges, the improvement of PCN production is very meaningful.

As described in previous studies, *Pseudomonas* species utilize conserved regulatory elements for phenazines production including the *gacS*/*gacA*, *rpeA/rpeB*, *psrA, pip*, *rpoS,* and the quorum sensing system *phzI*/*phzR*. The synthesis of core phenazine PCA is encoded by a conserved series of *phzABCDEFG* in phenazine-producing bacteria. In *P. aeruginosa* PAO1, *phzM*/*phzS*, *phzS,* and *phzH* genes encode typical enzymes involving in the transformation of PCA to pyocyanin (PYO), 1-hydroxy-phenazine (1-OH-PHZ), and PCN [40]. The conversion of PCA to PCN is catalyzed by *PhzH*, which was homologous with asparagine synthetases belonging to the class II glutamine amidotransferases. The sequence of *phzH* in strain HT66 is 99% similar to the *phzH* sequence of strain PCL1391. At the similar HPLC assays, little PCA and relatively large amount of PCN can be detected from 12  h fermentation broth. However, after 24 h of fermentation, only PCN can be found from the crude broth, which demonstrated the high efficiency of *phzH* in *P. chlororaphis* HT66 (Additional file 1: Figure S1).

Investigation of phenazine metabolic pathways provides critically important evidence for future genes manipulation so as to produce more phenazine derivatives for industrial usage. In this context, extensive research efforts have been devoted to understanding the phenazine-regulating mechanism. Herein, we investigated the role of two genes, *psrA* and *rpeA* on the phenazines biosynthesis. Both of these genes are sensitive to environmental changes and play a major role in determining phenazine production. To date, only two possible negative regulators of phenazine biosynthesis in *P. chlororaphis* are reported: *rpeA* [43] and *psrA* [27]. In strain 30–84, *rpeA* appears to markedly repress PCA biosynthesis in minimal medium. The PCA and 2-OH-PHZ production were considerably increased in *rpeA* inactivated GP72AN strain [38]. However, no report has been presented about the role of *psrA* and *rpeA* on the regulation of PCN biosynthesis. In this study, single mutant strains HT66Δ*psrA* and HT66Δ*rpeA* have been shown to produce about 1.66 and a 3.06-fold increase of PCN compared with the wild-type strain. On the other hand, a 4.24-fold enhancement in PCN production was recorded in double mutant HT66Δ*psrA*Δ*rpeA*, indicating that both *psrA* and *rpeA* are repressors for PCN biosynthesis in strain HT66.

**Fig. 6:**
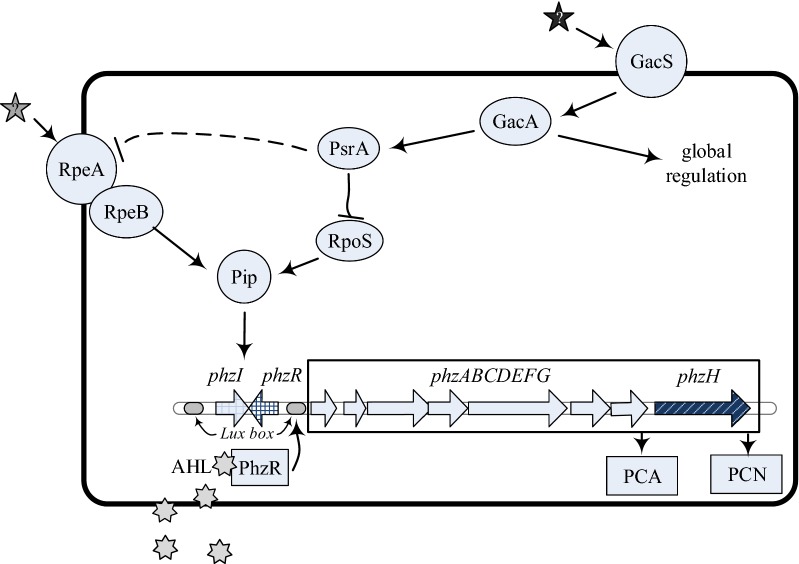
Hypothetical regulatory network for PCN biosynthesis in *Pseudomonas chlororaphis* HT66, The triangular arrowheads indicate a positive regulation, whereas the flat ones indicate a negative regulation. The proposed model shows the relationship between the *rpeA/rpeB* and *psrA* in relation to other known regulators. Finally, another important cascade must exist downstream of *psrA*. A to G, *phzA* to *phzG* genes; AHL, acyl-l-homoserine lactone

The qRT-PCR results showed a significantly increased level of PCN production in all the mutants (Table 2) that might be attributed to the high transcription levels of *phzI*, *phzR*, and other phenazine biosynthetic genes. The transcription level of *rpeA* and *rpeB* in strain HT66Δ*psrA* increased at 3.52- and 11.58-folds, respectively, indicating that *psrA* might act on *rpeA*/*rpeB* to regulate phenazine biosynthetic operon. The reduced *psrA* expression in HT66Δ*rpeA* evidenced a complex regulation mechanism for PCN production in HT66. Previous studies reported that *PsrA* binds to a *PsrA*-binding box to regulate *rpoS* in other *Pseudomonas* species [26]. The present findings show that *psrA* contributes to an additional level of regulation in strain HT66 by suppressing *phzI*/*phzR* or quorum sensing-regulated genes. One tentative proposal might be that the increased expression of *rpeA*/*rpeB* in HT66Δ*psrA* is a consequence of *psrA* regulating not only *rpoS* but also the *rpeA*/*rpeB* system, which influences indirectly the phenazine biosynthetic operon. Support for this interpretation comes from a putative theory which suggests that in *P. chlororaphis* PCL1391, the production of PCN differs little from wild-type after double mutation of *psrA*/*phzI* or *psrA*/*phzR*, whereas the PCN production cannot be detected in simply-mutated *phzI* or *phzR*. Conversely, high production of phenazine autoinducers not increases the PCN production compared with wild-type in the *psrA*/*phzR* double mutation strain PCL1391. All of these facts indicate that *psrA* might interact with a quorum sensing-independent regulator, which is more likely *rpeA,* to control PCN production [27, 28]. The little change of *rpoS* and *pip* transcription level in strain HT66Δ*psrA* may suggest that *psrA* mainly controls the *rpeA*/*rpeB* system to influence the PCN synthase in strain HT66 (Fig. 6).

The proposed model displays the relationship between the *rpeA/rpeB* and *psrA* in relation to other known regulators. Finally, another important cascade must exist downstream of *psrA*. Hence, this cascade involves the *rpeA/rpeB* (dotted lines) can be hypothesized. While the expression level of *psrA* in HT66Δ*rpeA* was reduced, taking into account the role of *psrA* and *rpeA* mutants are all environmentally dependent, the autoregulation of *psrA* might be affected by *rpeA*/*rpeB* system. The earlier study speculated that *rpeA*/*rpeB* can regulate the expression of a second quorum-sensing system except only *phzI*/*phzR* to control phenazine biosynthetic operon in *P. chlororaphis* 30–84. Phenazines production in the *phzR*/*rpeA* double mutant strain 30–84 was not different from the wild-type, as consistent with the conclusion that *rpeA*/*rpeB* system is regulated by *psrA* [31, 43]. Future research studies should be focused on the analysis of the interaction between *rpeA*/*rpeB* system and *psrA*. The role of different environmental conditions on the expression of *psrA*/*rpeA* and other related phenazine-regulating genes also need to be explained further in *P. chlororaphis* HT66.

## Conclusions

In this study, the novel strain HT66 of *P. chlororaphis* was isolated and identified through 16S rDNA sequence and cellular fatty acid analyses. The antifungal compound produced was purified and identified as PCN through mass spectrometry, and ^1^H, ^13^C nuclear magnetic resonance spectrum. Moreover, the newly isolated strain produced a high level of PCN with the fermentation titers of 1800.54 mg/L (4.24-fold increase) in double mutant strain HT66Δ*psrA*Δ*rpeA* compared with the wild-type HT66 that only produced 420.0 mg/L. In conclusion, high PCN biosynthesis by engineered *P. chlororaphis* HT66 demonstrates its great biotechnological perspective to hyperproduce not only PCN but also other valuable bio-pesticides for agricultural applications.

## Additional file

**Additional file 1.** Additional figures and tables.

## Abbreviations

PCN: phenazine-1-carboxamide
PGPR: plant growth-promoting rhizobacteria
PCA: phenazine-1-carboxylic acid
MFC: microbial fuel cells
2-OH-PHZ: 2-hydroxy-phenazine
AHL: *N*-acyl homoserine lactones
HPLC: high performance liquid chromatography
KB: King’s B medium
LB: Luria–Bertani
NMR: nuclear magnetic resonance
UMS: ultra-performance liquid and quadrupole time-of-flight mass spectrometry
Ct: threshold cycle
PYO: pyocyanin
1-OH-PHZ: 1-hydroxy-phenazine
