# Supplementary material for: Enhanced biosynthesis of phenazine-1-carboxamide by engineered Pseudomonas chlororaphis HT66
Source: Microb Cell Fact. 2018 Jul 25;17:117. doi: 10.1186/s12934-018-0962-3 (PMC6060551; doi:10.1186/s12934-018-0962-3)
Supplement: Supplementary file 1 — Additional file 1. Additional figures and tables. [file 12934_2018_962_MOESM1_ESM.docx]

**Additional file**

### Enhanced biosynthesis of phenazine-1-carboxamide by engineered *Pseudomonas chlororaphis* HT66

Huasong Peng^1^*, Pingyuan Zhang^1^, Muhammad Bilal^2^, Wei Wang^1^, Hongbo Hu^1,3^, Xuehong Zhang^1^

^1^State Key Laboratory of Microbial Metabolism, School of Life Sciences and Biotechnology, Shanghai Jiao Tong University, 800 Dongchuan Road, Shanghai 200240, People’s Republic of China

^2^School of Life Science and Food Engineering, Huaiyin Institute of Technology, Huaian 223003, China

^3^National Experimental Teaching Center for Life Sciences and Biotechnology, Shanghai Jiao Tong University, Shanghai, 200240, China

*Corresponding author e-mail: [hspeng@sjtu.edu.cn](mailto:hspeng@sjtu.edu.cn); Tel.: +86 021 3420 7047; Fax +86 021 3420 5081


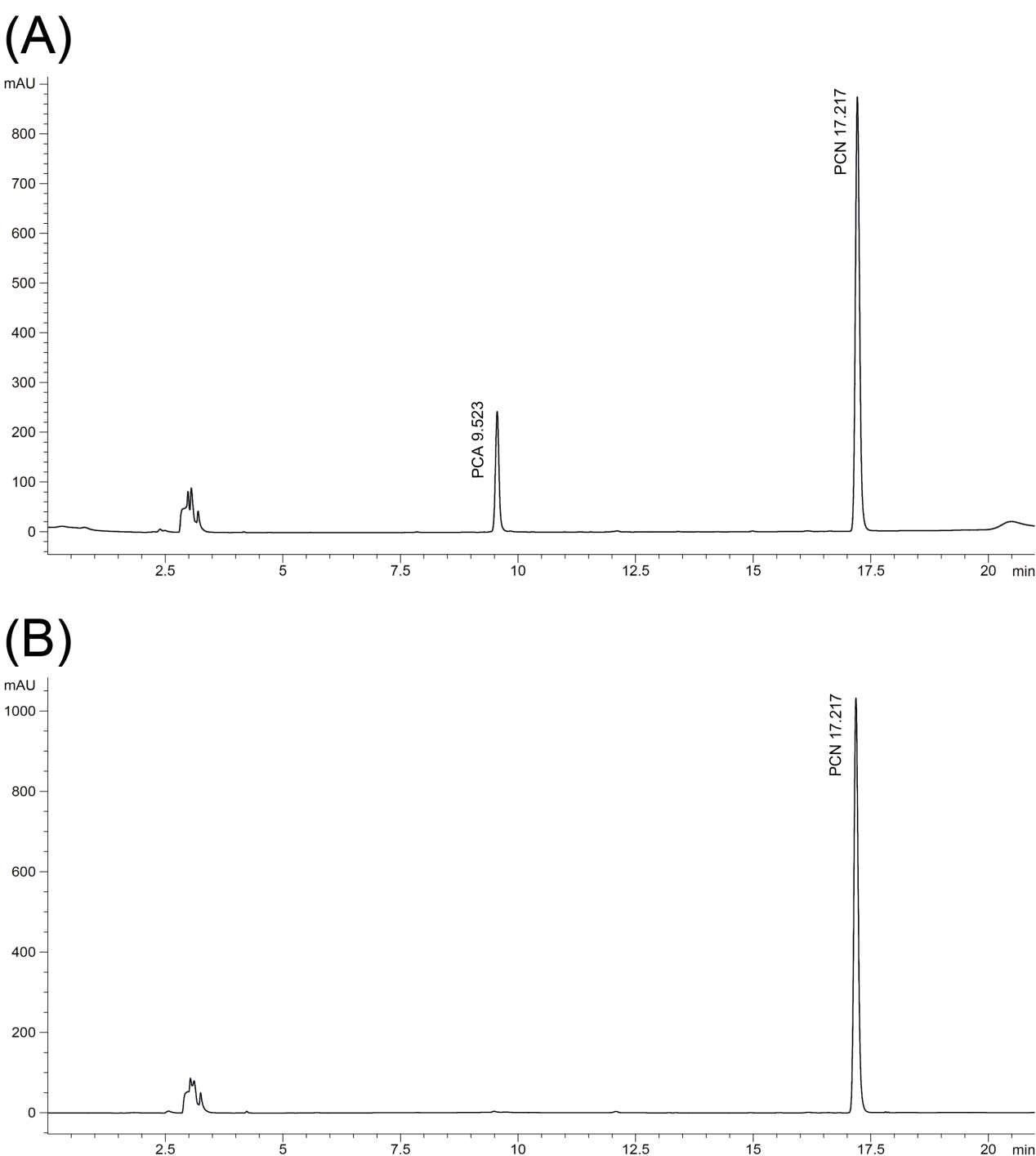


Figure S1 HPLC analysis of *Pseudomonas chlororaphis* HT66 culture after A) 12 and B) 24 h fermentation


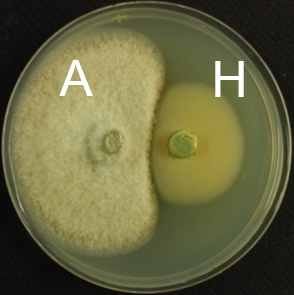

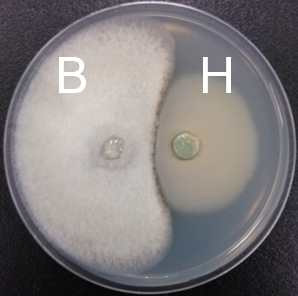

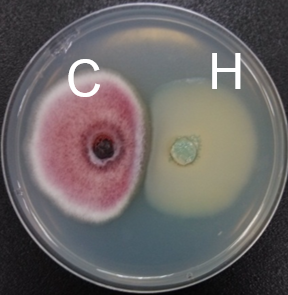


Figure S2 Inhibition of the growth of various fungi by strain HT66 (A: *Rhizoctonia solani*; B: *Pythium ultimum*; C: *Fusarium oxysporum*; H: *Pseudomonas chlororaphis* HT66)

Figure S3 Gas chromatography profile of FAME of strain HT66

Table S1 Oligonucleotides used for gene cloning and qPCR

| Oligonucleotide^a^ | Sequence (5’-3’) |
| --- | --- |
| *psrA*1 | TTAAGCTTACGGTCGGGTCGTCGCTGCATA (*HindIII*) |
| *psrA*2 | AAGAGCTCCGCTGTTCATGCCACGGATAAAG (*SacI*) |
| *rpeA*E1 | TATTAATCTAGACCTGTTCAGCCGTTCCGAAT (*XbaI*) |
| *rpeA*E2 | AATTATGAATTC-CCACGCCCAGTTGATCCT (*EcoRI*) |
| pBS-KAN-1 | TATAGCATGCTCAACCGTGGCTCCCTCA (*SphI*) |
| pBS-KAN-2 | TTAGCATGCCTTACTGTCATGCCATCCG (*SphI* ) |
| Gm-R & Gm-F | AGAATCGATATCCCCGGGTACCGAG (*ClaI*) |
| *gacS*RT1 | AAAACCTGGAAACCATCGAG |
| *gacS*RT2 | GGTGGGTGAAACCGAGGATG |
| *gacA*RT1 | GCGGCGGGTTACCTGACCAA |
| *gacA*RT2 | CATCTTGACGTCCATGAGGA |
| *psrA*RT1 | TCATTTCGGTTCCAAGAAGG |
| *psrA*RT2 | GACGAGGATTTCCAGCAGTT |
| *rpoS*RT1 | ATCAGTGGCTTTCCGAATTG |
| *rpoS*RT2 | GACCTTCGACCTGGATCTGG |
| *rpeA*RT1 | GCACCGCCATCTGCTCGTCC |
| *rpeA*RT2 | GCTGCACATCGTGCTGACCC |
| *rpeB*RT1 | CATCCTTCTGGTCGAAGACG |
| *rpeB*RT2 | AGGTCGAGAATCACCAGGTC |
| *pip*RT1 | AAAAGACCCGCGAGAACATT |
| *pip*RT2 | ACGTACAGCTGTTCCTTGCT |
| *phzI*RT1 | CTACCTCCTGGCGTTCAATG |
| *phzI*RT2 | GAAACGAGTCATTTCCCAGA |
| *phzR*RT1 | CGCAAGGACAACCCCATCAG |
| *phzR*RT2 | CACATTCCCTCCCGCTGAAC |
| *phzB*RT1 | CGCCGTCATGAACTATTTAC |
| *phzB*RT2 | GACGTTGTACCACTCCCAGT |
| 16SRT1 | ACGTCCTACGGGAGAAAGC |
| 16SRT2 | CGTGTCTCAGTTCCAGTGTGA |

^a^Underlined nucleotides are restriction sites added and the restriction enzymes are indicated at the end of primers

Table S2 Typical chromatogram and MIS report from FAME analysis of strain HT66

| RT | Response | Ar/Ht | RFact | ECL | Peak Name | Percent | Comment1 | Comment2 |
| --- | --- | --- | --- | --- | --- | --- | --- | --- |
| 1.0435 | 2035 | 0.017 | 1.178 | 9.0034 | 9:0 | 0.47 | ECL deviates 0.003 | Reference -0.006 |
| 1.1855 | 9828 | 0.012 | 1.133 | 9.9962 | 10:0 | 2.16 | ECL deviates -0.004 | Reference -0.013 |
| 1.4732 | 49697 | 0.011 | 1.074 | 11.4572 | 10:0 3OH | 10.37 | ECL deviates 0.009 |  |
| 1.5956 | 13358 | 0.017 | 1.054 | 11.9978 | 12:0 | 2.74 | ECL deviates -0.002 | Reference -0.011 |
| 1.6268 | 448 | 0.011 | 1.050 | 12.1177 | 11:0 iso 3OH | 0.09 | ECL deviates 0.010 |  |
| 1.9190 | 23202 | 0.012 | 1.015 | 13.2076 | 12:0 2OH | 4.57 | ECL deviates 0.004 |  |
| 1.9554 | 17604 | 0.011 | 1.011 | 13.3323 | 12:1 3OH | 3.46 | ECL deviates 0.007 |  |
| 2.0014 | 36309 | 0.010 | 1.006 | 13.4901 | 12:0 3OH | 7.10 | ECL deviates 0.007 |  |
| 2.1507 | 3990 | 0.012 | 0.992 | 14.0028 | 14:0 | 0.77 | ECL deviates 0.003 | Reference -0.006 |
| 2.6256 | 9829 | 0.012 | 0.957 | 15.5203 | Sum In Feature 2 | 1.83 | ECL deviates 0.005 | 14:0 3OH/16:1 iso I |
| 2.7279 | 105513 | 0.012 | 0.951 | 15.8411 | Sum In Feature 3 | 19.49 | ECL deviates 0.001 | 16:1 w7c/16:1 w6c |
| 2.7794 | 142912 | 0.012 | 0.948 | 16.0025 | 16:0 | 26.32 | ECL deviates 0.003 | Reference -0.008 |
| 2.9171 | 811 | 0.016 | 0.941 | 16.4319 | Sum In Feature 9 | 0.15 | ECL deviates -0.003 | 16:0 10-methyl |
| 2.9500 | 758 | 0.011 | 0.940 | 16.5346 | 15:0 3OH | 0.14 | ECL deviates 0.002 |  |
| 2.9830 | 816 | 0.015 | 0.938 | 16.6374 | 17:0 iso | 0.15 | ECL deviates 0.000 | Reference -0.010 |
| 3.0467 | 848 | 0.013 | 0.935 | 16.8362 | 17:1 w7c | 0.15 | ECL deviates 0.000 |  |
| 3.0719 | 56398 | 0.012 | 0.934 | 16.9148 | 17:0 cyclo | 10.24 | ECL deviates 0.000 |  |
| 3.0982 | 1039 | 0.012 | 0.933 | 16.9969 | 17:0 | 0.19 | ECL deviates -0.003 | Reference -0.014 |
| 3.2757 | 1818 | 0.013 | 0.928 | 17.5545 | 16:0 3OH | 0.33 | ECL deviates 0.006 |  |
| 3.3402 | 1426 | 0.013 | 0.926 | 17.7572 | Sum In Feature 5 | 0.26 | ECL deviates 0.001 | 18:2 w6,9c/18:0 ante |
| 3.3684 | 46803 | 0.012 | 0.925 | 17.8457 | Sum In Feature 8 | 8.41 | ECL deviates -0.002 | 18:1 w7c |
| 3.4175 | 1914 | 0.013 | 0.924 | 18.0001 | 18:0 | 0.34 | ECL deviates 0.000 | Reference -0.012 |
| 3.7070 | 864 | 0.012 | 0.920 | 18.9299 | 19:0 cyclo w8c | 0.15 | ECL deviates -0.002 |  |
| 3.7280 | 793 | 0.012 | 0.920 | 18.9974 | 19:0 | 0.14 | ECL deviates -0.003 |  |
